# Supplementary material for: A peridomestic Aedes malayensis population in Singapore can transmit yellow fever virus
Source: PLoS Negl Trop Dis. 2019 Oct 7;13(10):e0007783. doi: 10.1371/journal.pntd.0007783 (PMC6797215; doi:10.1371/journal.pntd.0007783)
Supplement: Attachment — Submitted filename: YFV Ae. malayensis Singapore final.docx [file pntd.0007783.s001.docx]

**PNTD-D-19-01129**

**A Peridomestic *Aedes malayensis* Population Increases Risk of Yellow Fever Virus Transmission in Singapore**

**General comments:**

This manuscript reports experiments conducted to assess the vector competence of a Singapore Aedes malayensis for an African strain of yellow fever virus. Key measures of vector competence were similar to *Aedes aegypti*, the primary characterized urban vector of the virus. The authors suggest that this increases the threat of yellow fever virus transmission in Singapore. Given the recent re-emergence of yellow fever in Africa and South America, this work is of relevance to readers of PLoS Neglected Tropical Diseases and potentially warrants publication once the comments listed below have been addressed.

**Major comments:**

- The presence of Ae. malayensis does not increase the risk of YFV transmission in Singapore. This suggests that the risk was lower previously, which it probably was. However, given that this species has always been present, its contribution would have remained the same and other factors are leading to an increase in risk. Instead, this species could contribute to the risk or contribute to transmission cycles. The title and text should be modified to show this.
- The manuscript describes experiments conducted on *Ae. malayensis* as a peridomestic species. However, there is next to nothing mentioned about *Ae. albopictus*, which is also a peridomestic species that is highly prevalent in Singapore. Indeed, this species accounted for the highest proportion of mosquitoes attracted to the human bait. Thus, the current manuscript needs to discuss the results in the context of *Ae. albopictus* sharing a similar niche to *Ae. malayensis*, and, to a lesser degree, with *Ae. aegypti*.
- The host-seeking behavior experiments are too preliminary in nature to be included in the manuscript text. As reported, they were not conducted in a rigorous scientific manner (no replication, no controls, no information on how many days samples were taken etc.). This aspect of the paper should be provided as supplemental material.
- Mention that *Ae. malayensis* is a member of the *Stegomyia* sub-genus, which is the sub-genus that *Ae. aegypti* and other YFV vectors belong to.
- Finally, provide a sentence (or two) discussing possible reasons that YFV has not caused outbreaks in SE Asia.

**Minor comments (numbers refer to the line/s in the manuscript draft):**

29-30: Also mention that the risk is higher due to recent epidemic activity in Africa and South America.

30: Change “higher” to “high”.

32: State what arboviruses have re-emerged.

37: Need to provide some measures of vector competence (i.e. infection rates, transmission rates). Were levels of vector competence between the 2 species not significant?

65: Are the 2016 cases in China really the first documented cases of YFV in Asia? If so, it would be more prudent to say they were the first cases diagnosed in travellers to Asia. Also, it should read “11 Chinese workers infected with YFV in Angola who returned to China…”.

65-67: Also mention that mosquito control programs would be overwhelmed.

72-73: Delete parentheses

97-99: Provide the generation number for the *Ae. malayensis* colony used in the experiments.

113: Where did the patient return to?

121: You don’t know that mosquitoes are infected until they are analyzed at a later stage. Change this to “Virus Challenge” or “Exposure of mosquitoes to YFV”.

133: What is the container volume in mL?

137-138: Why were legs and wings removed in one experiment and heads in the other? Also, were these body components placed in some form of growth medium before storages at -80^o^C?

144-150: Move the section on vector competence parameters to the statistical analyses section. Then rename this section “Virus detection” or “Virus analysis”, as that is what this paragraph is really about.

152: Should it be “homogenization buffer” and not “squash buffer”?

155-157: Is this a published method? If so, provide the reference. It is cited later, but here would be more appropriate.

165-167: Provide some justification as to why wings/legs and heads were processed using the FFA, whilst bodies were processed with RT-PCR.

186-188: Reword. It sounds like the sticky inner surface is the oviposition site.

195-196: Why was the analysis of dissemination and transmission done only on day 14?

259-260: Emphasise that results for *Ae. malayensis* were not significantly different from *Ae. aegypti*.

261: Acronyms should be used for virus names after first use.

264-267: The results of the current study do not support the statement that anthropophilic. Human was the only bait used. If other animals were offered and they stiff chose human, then this conclusion could be drawn. Need to say that the current study provides evidence that *Ae. malayensis* will bite humans.

270-217: Reword to say that it is prevalent in numerous urban parks of Singapore.

271-273: Provide reference for this.

282: Provide an example of a natural larval habitat in Singapore.

282-283. Delete the sentence on application of pyriproxyfen. This is only one of several control options available.

292: Other factors can also impact comparisons between studies, such as the method of virus assay, strain of mosquitoes used for the experiments etc.

294-297: This really highlights the threshold for infection, so this should be mentioned.

299-301: Delete this sentence. It is confusing.

306: Should it be “rampant”?

310: Delete “spillback”.

471 and 480: State that the data from the two experiments is combined.
