# Supplementary material for: A peridomestic Aedes malayensis population in Singapore can transmit yellow fever virus
Source: PLoS Negl Trop Dis. 2019 Oct 7;13(10):e0007783. doi: 10.1371/journal.pntd.0007783 (PMC6797215; doi:10.1371/journal.pntd.0007783)
Supplement: Attachment — Submitted filename: Miot_response_to_reviewers.docx [file pntd.0007783.s002.docx]

Paris, 12 September 2019

Dear Dr. Viennet,

Thank you for your correspondence about our manuscript PNTD-D-19-01129 entitled “A peridomestic *Aedes malayensis* population increases risk of yellow fever virus transmission in Singapore".

We are submitting a revised version of our manuscript in which we addressed the Reviewers’ comments. The revisions are detailed in the point-by-point response and in the enclosed version of the manuscript with tracked changes.

## Please note that following the Reviewers’ comments, we have changed the title to “A peridomestic *Aedes malayensis* population in Singapore can transmit yellow fever virus”.

## We thank the reviewers for their insights, which contributed to greatly improve the manuscript.

Please let me know if you need any additional information.

Thank you very much for your consideration.

Sincerely,

Louis Lambrechts

Head, Insect-Virus Interactions Unit

Department of Virology

Institut Pasteur – CNRS UMR 2000

28 rue du Docteur Roux

75724 Paris Cedex 15 FRANCE

**Reviewer #1:**

This study seeks to clarify the degree to which occurrence of Aedes malayensis in Singapore exacerbates the risk of yellow fever virus introduction to that country.  The authors conducted a rigorous experimental study of vector competence with an Aedes aegypti control, and a field study to ascertain human attraction.  It is the latter that is tripping me up a little, in that no unbaited traps were used to determine the number of Ae. malayensis that might have entered the net in the course of daily movement.  Can the authors specify what the "other" mosquitoes captured via this method were?  Were any of them species well known to not be attracted to humans?

*Response*: We agree that our host-seeking experiment was small scale and lacked a non-baited trap as a control. Accordingly we toned down our conclusions on *Ae. malayensis* host-seeking behavior and removed the term “anthropophilic” throughout the paper. As shown in Fig. 3B, the majority (89%) of mosquitoes that entered the human-baited trap were *Ae. albopictus*, a species well known for its human-biting behavior.

Statistical analyses are rigorous but I would ask that the authors delete text referring to "marginally insignificant" results and remove symbols to denote "P

*Response*: As per the Reviewer’s request we removed the sentence referring to “a marginally insignificant effect” and the statistical significance symbols in Table 1.

I am somewhat concerned about drawing strong conclusions about human attraction from the field data without an unbaited control; the authors could allay this concern if there are studies in the literature showing a clear difference. Also, the authors should discuss the capture of a large number of Ae. albopictus in this urban park. This species has also been shown to be competent for YFV and to my mind poses the larger risk here.

*Response*: As mentioned above, we toned down our conclusions on *Ae. malayensis* host-seeking behavior in Singapore and removed the term “anthropophilic” throughout the paper. We also added a sentence to emphasize the risk of arbovirus transmission associated with *Ae. albopictus* in the discussion.

Throughout, including title: Change to "Occurrence of a peridomestic Aedes malayensis population..." It is not the global existence of Ae. malayensis that exacerbates risk in Singapore, it is the occurrence of this species IN Singapore that exacerbates risk there.

*Response*: We changed the title to “A peridomestic *Aedes malayensis* population in Singapore can transmit yellow fever virus” and do not refer to the risk of YFV transmission any more.

Line 30: Higher than what? Clarify

*Response*: We changed “higher” to “high”.

Line 38: Change "a" to "one" and indicate 3 trap days- this was a very limited study and that should be made more clear.

*Response*: We changed the abstract sentence to “In addition, we captured *Ae. malayensis* females in one human-baited trap during three days of collection, providing preliminary evidence that host-vector contact may occur in field conditions.”

Line 50: move "was" before "Ae. malayensis"

*Response*: Done.

Line 51: move "experimentally" after "to"- infinitives may be split when necessary for clarity!

*Response*: Done.

Summary: reconsider what has actually been demonstrated about human attraction based on comments above

*Response*: We emphasized the preliminary nature of our evidence and referred to host-vector contact instead of anthropophilic behavior.

Line 66: delete "largely"

*Response*: Done.

Line 122: Change thereafter to hereafter

*Response*: Done.

Line 177: In future, please provide volunteers with a motorized aspirator to enhance safety

*Response*: Noted.

Throughout: the term "insignificant" is not standard; please change to "not significant"

*Response*: Done.

Line 271-272: move "were" to after "only"

*Response*: Done.

Table 1: Please reformat to improve legibility (font is variable and some of it tiny); remove annotation of P

*Response*: Done.

**Reviewer #2:**

In this excellent study, the investigators have demonstrated that Ae. malayensis, an anthpophilic mosquito in Singapore, is a competent vector of YFV. Ae. malayensis occurs in some urban habitats that are not currently targeted in Ae. aegypti control programs, and they may contribute to “cryptic” arbovirus (DENV, CHIKV, ZIKV) transmission. But these findings do not increase the risk of YFV transmission in Singapore.

Ae. malayensis was first described in Singapore in 1962 and has subsequently been reported in Malaysia, Thailand, Cambodia, Vietnam and Taiwan. It probably occurs throughout southeastern Asia. So it is not a new introduction and has probably been in the region for thousands of years. Given that history and the multiple opportunities for introduction of YFV into the region in the past, is Singapore now at greater risk of a yellow fever outbreak that it was 200 or 400 years ago? If Ae. aegypti populations are maintained at a low level in Singapore, it may actually be at a lower risk, regardless of the presence of Ae. malayensis.

In the opinion of this reviewer, a more appropriate title for this article would be something like this: “Aedes malayensis, a peridomestic mosquito in Singapore, is a competent vector of yellow fever virus.” In the discussion, the authors could speculate that this finding might facilitate the introduction of YFV into Singapore or other countries in the region where Ae. malayensis occurs.

*Response*: We changed the title to “A peridomestic *Aedes malayensis* population in Singapore can transmit yellow fever virus” and do not refer to the risk of YFV transmission any more. We also added a sentence in the discussion to indicate that competent mosquito species such as *Ae. malayensis* could facilitate introduction of YFV into the Asia-Pacific region.

**Reviewer #3:**

Major comments:

- The presence of Ae. malayensis does not increase the risk of YFV transmission in Singapore. This suggests that the risk was lower previously, which it probably was. However, given that this species has always been present, its contribution would have remained the same and other factors are leading to an increase in risk. Instead, this species could contribute to the risk or contribute to transmission cycles. The title and text should be modified to show this.

*Response*: We changed the title to “A peridomestic *Aedes malayensis* population in Singapore can transmit yellow fever virus” and do not quantify the contribution of *Ae. malayensis* to the risk of YFV transmission any more. We made further edits in the abstract and introduction to clarify this point.

- The manuscript describes experiments conducted on *Ae. malayensis* as a peridomestic species. However, there is next to nothing mentioned about *Ae. albopictus*, which is also a peridomestic species that is highly prevalent in Singapore. Indeed, this species accounted for the highest proportion of mosquitoes attracted to the human bait. Thus, the current manuscript needs to discuss the results in the context of *Ae. albopictus* sharing a similar niche to *Ae. malayensis*, and, to a lesser degree, with *Ae. aegypti*.

*Response*: We added a sentence in the discussion to emphasize the risk of arbovirus transmission associated with *Ae. albopictus*.

- The host-seeking behavior experiments are too preliminary in nature to be included in the manuscript text. As reported, they were not conducted in a rigorous scientific manner (no replication, no controls, no information on how many days samples were taken etc.). This aspect of the paper should be provided as supplemental material.

*Response*: We agree that our host-seeking experiment was small scale and lacked a non-baited trap as a control. Accordingly we toned down our conclusions on *Ae. malayensis* host-seeking behavior. We removed the term “anthropophilic” throughout the paper and referred to host-vector contact instead. We leave it to the discretion of the editor but we believe that the field-based evidence deserves to remain in the main body of the paper provided its limitations are clearly presented. The abstract now states “we captured *Ae. malayensis* females in one human-baited trap during three days of collection, providing preliminary evidence that host-vector contact may occur in field conditions.”

- Mention that *Ae. malayensis* is a member of the *Stegomyia* sub-genus, which is the sub-genus that *Ae. aegypti* and other YFV vectors belong to.

*Response*: Done.

- Finally, provide a sentence (or two) discussing possible reasons that YFV has not caused outbreaks in SE Asia.

*Response*: We added the following sentence in the discussion: “The lack of YFV outbreaks in the Asia-Pacific region to date could be due to cross-protective immunity conferred by other widespread flaviviruses such as DENV and Japanese encephalitis virus, but it could also reflect relatively fewer opportunities for YFV introduction until now.”

Minor comments

29-30: Also mention that the risk is higher due to recent epidemic activity in Africa and South America.

*Response*: We changed the sentence to “the risk of introduction has never been higher due to the increasing influx of people from endemic regions and recent outbreaks in Africa and South America.”

30: Change “higher” to “high”.

*Response*: Done.

32: State what arboviruses have re-emerged.

*Response*: We specified re-emergence of dengue, chikungunya and Zika viruses.

37: Need to provide some measures of vector competence (i.e. infection rates, transmission rates). Were levels of vector competence between the 2 species not significant?

*Response*: The various vector competence phenotypes and their statistical analysis are described in detail in the main body of the manuscript. With all due respect, we feel that these details do not belong to the abstract.

65: Are the 2016 cases in China really the first documented cases of YFV in Asia? If so, it would be more prudent to say they were the first cases diagnosed in travellers to Asia. Also, it should read “11 Chinese workers infected with YFV in Angola who returned to China…”.

*Response*: This sentence was modified as per the Reviewer’s suggestions.

65-67: Also mention that mosquito control programs would be overwhelmed.

*Response*: Done.

72-73: Delete parentheses

*Response*: Done.

97-99: Provide the generation number for the *Ae. malayensis* colony used in the experiments.

*Response*: We indicated that the number of generations is unknown but >50.

113: Where did the patient return to?

*Response*: We specified that the patient returned to France.

121: You don’t know that mosquitoes are infected until they are analyzed at a later stage. Change this to “Virus Challenge” or “Exposure of mosquitoes to YFV”.

*Response*: We changed the subheading to “Virus Challenge”.

133: What is the container volume in mL?

*Response*: We changed to “1-pint” to “0.5-L”.

137-138: Why were legs and wings removed in one experiment and heads in the other? Also, were these body components placed in some form of growth medium before storages at -80^o^C?

*Response*: We changed methodology between the two experiments because the triethylamine protocol to perform *in vitro* salivation was implemented in the meantime. Mosquitoes were only paralyzed with triethylamine in the second experiment. Removing heads is easier than removing legs and wings but could not be done in the first experiment because the mosquitoes were non-anesthetized during saliva collection. We specified that the body components were stored dry.

144-150: Move the section on vector competence parameters to the statistical analyses section. Then rename this section “Virus detection” or “Virus analysis”, as that is what this paragraph is really about.

*Response*: Done.

152: Should it be “homogenization buffer” and not “squash buffer”?

*Response*: We changed “squash buffer” to “lysis buffer”.

155-157: Is this a published method? If so, provide the reference. It is cited later, but here would be more appropriate.

*Response*: This method is unpublished with the exception of the primers.

165-167: Provide some justification as to why wings/legs and heads were processed using the FFA, whilst bodies were processed with RT-PCR.

*Response*: Infected bodies were screened by RT-PCR, which detects viral RNA, but for dissemination we chose to use an infectious assay because the amount of viral RNA does not necessarily reflect the amount of infectious particles. This is because dissemination titer would have been a backup phenotype should the saliva titration have failed.

186-188: Reword. It sounds like the sticky inner surface is the oviposition site.

*Response*: We changed the sentence to “The Gravitrap is a simple, hay infusion-filled cylindrical trap with a sticky inner surface to catch ovipositing gravid female *Aedes* mosquitoes.”

195-196: Why was the analysis of dissemination and transmission done only on day 14?

*Response*: We specified that “the analysis of dissemination and transmission rates was restricted to 14 days post blood meal because the two time points differed and there was too little data on day 10 for a meaningful analysis.”

259-260: Emphasise that results for *Ae. malayensis* were not significantly different from *Ae. aegypti*.

*Response*: We changed the sentence to “our laboratory mosquito colony recently derived from a wild *Ae. malayensis* population in Singapore was experimentally competent for YFV, to a level that was not statistically different from that of the *Ae. aegypti* controls.”

261: Acronyms should be used for virus names after first use.

*Response*: Done.

264-267: The results of the current study do not support the statement that anthropophilic. Human was the only bait used. If other animals were offered and they stiff chose human, then this conclusion could be drawn. Need to say that the current study provides evidence that *Ae. malayensis* will bite humans.

*Response*: We changed this sentence to “Although our host-seeking experiment lacked a non-baited control trap, it provided preliminary evidence that this peridomestic *Ae. malayensis* population in Singapore may engage in human-biting behavior, as was already observed for other populations of the species in South-East Asia.”

270-217: Reword to say that it is prevalent in numerous urban parks of Singapore.

*Response*: Done.

271-273: Provide reference for this.

*Response*: Done.

282: Provide an example of a natural larval habitat in Singapore.

*Response*: We mentioned tree holes as natural breeding sites that are less accessible.

282-283. Delete the sentence on application of pyriproxyfen. This is only one of several control options available.

*Response*: Done.

292: Other factors can also impact comparisons between studies, such as the method of virus assay, strain of mosquitoes used for the experiments etc.

*Response*: We changed the sentence to “vector competence results cannot be readily compared if they did not use the same oral infectious dose, or if other factors such as virus strain and detection assays were different.”

294-297: This really highlights the threshold for infection, so this should be mentioned.

*Response*: We added the sentence “This example indicates that the minimal threshold for infection may differ between mosquito populations.”

299-301: Delete this sentence. It is confusing.

*Response*: To clarify, we rephrased this sentence to “In natural conditions, the oral infectious dose is the primary driver of successful human-to-mosquito transmission of arboviruses.”

306: Should it be “rampant”?

*Response*: Yes, thank you.

310: Delete “spillback”.

*Response*: Done.

471 and 480: State that the data from the two experiments is combined.

*Response*: Done.
